# Dynamics of the immune repertoire in recurrent, locally advanced NSCLC not amenable for definitive therapy and in stage IV disease receiving first-line chemotherapy

**DOI:** 10.3389/fonc.2026.1787457

**Published:** 2026-04-13

**Authors:** Narumol Trachu, Dulyathat Anantaya, Nareenart Iemwimangsa, Salin Amponnavarat, View-Hune Teoh, Songporn Oranratnachai, Khantong Khiewngam, Thanaporn Thamrongjirapat, Nanamon Monnamo, Putthapoom Lumjiaktase, Thitiya Dejthevaporn, Ekaphop Sirachainan, Wasun Chantratita, Thanyanan Reungwetwattana

**Affiliations:** 1 Offices of Health Science Research, Faculty of Medicine Ramathibodi Hospital, Mahidol University, Bangkok, Thailand; 2 Division of Medical Oncology, Department of Medicine, Faculty of Medicine Ramathibodi Hospital, Mahidol University, Bangkok, Thailand; 3 Faculty of Medicine Ramathibodi Hospital, Ramathibodi Lung Cancer Consortium (RLC), Mahidol University, Bangkok, Thailand; 4 Center for Medical Genomics, Faculty of Medicine Ramathibodi Hospital, Mahidol University, Bangkok, Thailand; 5 Chakri Naruebodindra Institute, Faculty of Medicine Ramathibodi Hospital, Mahidol University, Bangkok, Thailand; 6 Oncology Unit Sriphat Medical Center, Faculty of Medicine, Chiangmai University, Chiangmai, Thailand; 7 Department of Medicine, Faculty of Medicine Ramathibodi Hospital, Mahidol University, Bangkok, Thailand; 8 Department of Pathology, Faculty of Medicine Ramathibodi Hospital, Mahidol University, Bangkok, Thailand

**Keywords:** chemotherapy immunomodulation, clonal diversity, immune biomarker, non-small cell lung cancer, principal component analysis, Shannon diversity, TCR convergence, TCR repertoire

## Abstract

**Background:**

Cytotoxic chemotherapy can modulate antitumor immunity, yet its impact on the peripheral T-cell receptor (TCR) repertoire in non-targetable advanced NSCLC remains poorly characterized. We prospectively investigated chemotherapy-induced TCRβ repertoire dynamics and their prognostic relevance.

**Methods:**

Patients with recurrent unresectable locally advanced or stage IV NSCLC without actionable mutations received first-line platinum-based chemotherapy (no immunotherapy) at Ramathibodi Hospital (2021–2024). Peripheral blood was collected at baseline (T1), chemotherapy completion (T2), and confirmed disease progression (T3). TCRβ sequencing (Ion Torrent™ Oncomine™ TCR Beta-LR) was performed on samples rarefied to >1.5 million reads. Shannon diversity, Pielou evenness, TCR convergence frequency, unique clone counts, and principal component analysis (PCA) of clonal frequencies were analyzed. Multiple comparisons were Benjamini–Hochberg corrected (significance: p<0.05, BH-adj. q<0.05).

**Results:**

Of 42 enrolled patients, 34 were T1-evaluable and 15 at T2; longitudinal attrition was driven primarily by pre-T2 death (74% vs. 27%; p=0.014). Disease control was achieved in 11/15 T2-evaluable patients (73%). Disease control patients trended higher TCR convergence (0.0040 vs. 0.0023) and significantly smaller decline in unique clone counts (−16% vs. −41%; Wilcoxon p=0.020, q=0.030). PCA revealed compact repertoire clustering in disease control versus wide PC2 dispersion in progressive disease (PD) patients (p=0.030, q=0.030). Among 4 patients reaching T3 (all PD), post-chemotherapy rises in Shannon diversity and convergence reversed at T3, consistent with immune attrition. Overall survival was significantly longer in disease control patients (log-rank p=0.036).

**Conclusion:**

Stable clonal convergence and preserved clone counts associate with disease control and survival in non-targetable advanced NSCLC receiving chemotherapy, supporting peripheral TCRβ profiling as an exploratory biomarker warranting prospective validation.

## Background

Non–small cell lung cancer (NSCLC) is the leading cause of cancer mortality worldwide and accounts for approximately 85% of lung cancer diagnoses (1–3). Survival is strongly stage dependent; outcomes for advanced or metastatic disease remain poor (2, 3). In patients without actionable genomic alterations, cytotoxic chemotherapy has historically constituted standard management, with modest survival benefit and substantial toxicity. Over the past decade, immune checkpoint inhibitors (ICIs) directed at PD-1/PD-L1 and CTLA-4 have altered first-line and consolidation algorithms and can induce durable benefit in a subset of patients (4–9). Nevertheless, primary and acquired resistance are common, and predictive biomarkers with adequate performance across clinical contexts are limited.

High-throughput profiling of the T-cell receptor (TCR) repertoire provides quantitative readouts of treatment-perturbed adaptive immunity in NSCLC. Summary measures frequently used include Shannon diversity (repertoire breadth), Pielou evenness (uniformity of clonal frequencies), and TCR convergence (independent nucleotide rearrangements encoding identical CDR3 amino-acid sequences), a putative surrogate of antigen-driven selection (10–15, 23–25). Cross-sectional and longitudinal studies have associated these features with tumor biology and outcomes during ICI therapy, indicating that repertoire structure captures clinically relevant immune states that are incompletely reflected by static markers such as PD-L1 immunohistochemistry (10–12). While humoral components including B-cell and plasma-cell repertoires and tertiary lymphoid structures — also correlate with therapeutic responsiveness and interact with T-cell–mediated antitumor activity, characterization of these compartments was beyond the scope of the present study, which focuses on the TCRβ repertoire as the primary analyte (12, 16, 17).

Although biomarker development has emphasized immunotherapy cohorts, cytotoxic chemotherapy can modulate antitumor immunity. Several agents induce immunogenic cell death, increase antigen presentation, and alter dendritic and T-cell function (18–21). Within the cancer-immunity cycle, these effects could transiently broaden the circulating TCR repertoire and facilitate clonal priming (22). However, rigorous descriptions of dynamic repertoire remodeling during chemotherapy particularly in patients with non-targetable advanced NSCLC remain limited (3–6, 9). Because many patients present without targetable drivers or develop resistance to targeted agents, defining immune kinetics under chemotherapy is clinically relevant and may inform sequencing or combination strategies (e.g., chemo-IO) designed to maintain productive clonal responses (6, 9, 18–22).

Dimensionality-reduction methods, including principal component analysis (PCA), are useful for summarizing high-dimensional clonal frequency matrices and for visualizing repertoire organization at the patient level. Prior work in thoracic oncology indicates that phenotypes associated with outcome often map to separations along leading principal components and to differences in dispersion or entropy, reflecting heterogeneous versus focused clonal expansion patterns (10, 15). Analyses that integrate classical indices (diversity, evenness) with convergence and multivariate structure are therefore likely to yield more informative biomarkers than any single metric.

Existing evidence supports the clinical relevance of these measurements. Comprehensive profiling in NSCLC has linked T-cell repertoire features to tumor biology and survival (10). In a consolidation-immunotherapy setting, increased TCR convergence correlated with disease control during durvalumab, consistent with expansion of tumor-reactive clonotypes (11). Population-level immunosequencing has further shown that repertoire diversity and clonal selection encode immunologic history and HLA context, supporting the biological plausibility of convergence as a readout of shared antigen recognition (14, 15). Observations from humoral compartments and lymphoid organization reinforce the broader concept that effective antitumor immunity is architecturally coordinated — a framework that motivates multivariate approaches such as the combined analysis of diversity, evenness, convergence, and PCA employed here (12, 16, 17).

These considerations suggest a working model for chemotherapy-treated NSCLC: initial treatment may broaden repertoire breadth, followed by either clonal focusing (in patients who achieve disease control) or dissipation/attrition (in those who progress) (18–22). Testing this model in non-targetable advanced NSCLC may extend the utility of repertoire-based biomarkers beyond immunotherapy alone and help identify patients most likely to benefit from specific systemic strategies.

We sought to characterize chemotherapy-induced TCR repertoire remodeling in non-targetable advanced NSCLC and evaluate its prognostic implications. Specifically, this study aimed to: (i) document longitudinal changes in peripheral TCRβ repertoires in patients with unresectable locally advanced or metastatic non-targetable NSCLC receiving first-line chemotherapy; (ii) compare trajectories of Shannon diversity, Pielou evenness, and TCR convergence between disease control and progressive disease (PD) patients; and (iii) determine whether multivariate clonal organization assessed by PCA provides additional prognostic information beyond univariate repertoire metrics.

## Methods

### Patient and study design

This was a prospective longitudinal cohort study conducted at Ramathibodi Hospital, Bangkok, Thailand, between November 2021 and January 2024. Eligible participants were patients aged 18 years or older with histologically confirmed non-small cell lung cancer (NSCLC), classified according to the 8th edition of the American Joint Committee on Cancer (AJCC) staging system. Patients were eligible if they had recurrent NSCLC after prior definitive local therapy (surgery or chemoradiation) with no further curative options, unresectable locally advanced NSCLC not amenable to surgery or concurrent chemoradiotherapy, or stage IV NSCLC at the time of diagnosis. All patients had either known or unknown non-targetable driver mutations and were receiving first-line systemic chemotherapy as standard of care. Immunotherapy was not permitted as part of the treatment regimen. The survival follow-up cut-off date was April 2024. Overall survival (OS) was defined as the time from the date of first-line chemotherapy initiation to death from any cause. Patients who were alive at the data cut-off date were censored at their last known date of contact. OS follow-up was conducted independently of blood sample collection and RNA quality; patients were followed for survival regardless of whether peripheral blood samples were available or evaluable at any given time point.

**Figure 1:**
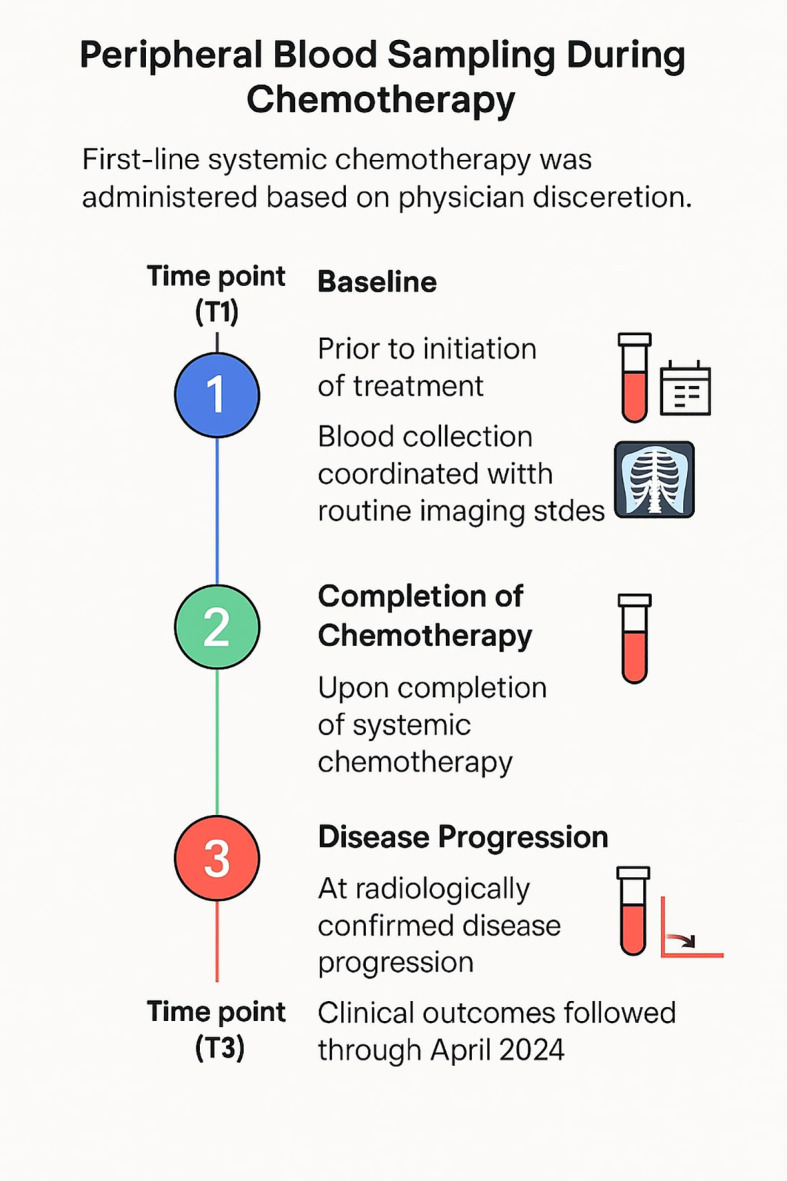
Immune repertoire blood sampling time points during chemotherapy.

Patients were excluded if they had received any prior systemic therapy for advanced disease, including chemotherapy, targeted therapy, or immunotherapy. Patients were excluded if tumor tissue or peripheral blood samples at baseline were inadequate for immune repertoire analysis due to insufficient DNA quality or quantity. Other exclusion criteria included the presence of concurrent malignancies apart from adequately treated non-melanoma skin cancers or *in situ* cervical carcinoma, uncontrolled comorbid conditions such as severe cardiovascular disease, active infections, or significant hepatic or renal dysfunction, and an ECOG performance status ≥3. Pregnant or breastfeeding women were not eligible, and patients with incomplete clinical data or follow-up records were also excluded.First-line systemic chemotherapy was administered based on physician discretion. Peripheral blood samples were collected at three defined time points: baseline (prior to initiation of treatment, Time point 1: T1), upon completion of systemic chemotherapy (Time point 2: T2), and at the time of radiologically confirmed disease progression (Time point 3: T3) (**Figure 1**). Blood collection was coordinated with routine imaging studies (chest X-ray or CT scan) used for standard clinical disease monitoring and continued until completion of chemotherapy or documentation of progression. Clinical outcomes, including disease progression and survival, were followed through April 2024, the data cut-off point. Treatment response was evaluated by the investigator according to the Response Evaluation Criteria in Solid Tumors (RECIST) criteria version 1.1.

Disease progression was defined according to RECIST version 1.1 criteria, including unequivocal progression of non-target lesions, a ≥20% increase in target lesion size, or the appearance of new lesions.

Peripheral blood was collected into EDTA-anticoagulated vacutainer tubes. Samples were processed within 2–4 hours of collection to preserve RNA integrity. Peripheral blood mononuclear cells (PBMCs) were isolated by density-gradient centrifugation at 400×g for 15 minutes at 4 °C. RNA was extracted from peripheral blood mononuclear cells using the MagMAX™ mirVana™ Total RNA Isolation Kit (Thermo Fisher Scientific, Waltham, MA, USA, catalog number A27828). RNA quality was assessed with the Agilent™ TapeStation RNA ScreenTape system(catalog number 5067-5579), which provided both concentration and integrity values. Only samples with adequate yield and RNA integrity number (RIN)-equivalent ≥7.0 were advanced for sequencing to ensure reliability of downstream analyses.

High-throughput TCRβ library preparation and sequencing were performed using the Ion Torrent™ Oncomine™ TCR Beta-LR (Long-Read) Assay (Thermo Fisher Scientific, catalog number A46297), targeting the CDR3 region of the TCRβ chain using multiplex PCR amplification. Sequencing was performed on the Ion Torrent S5™platform. To minimize inter-run technical variability, samples from the same individual collected at different time points were processed within the same sequencing batch wherever feasible. Sequencing runs were randomized with respect to clinical outcome group allocation. Internal negative controls were included in each run to monitor contamination.

Data processing and clonotype assignment were performed using Ion Reporter™ Software (Thermo Fisher Scientific). To minimize batch effects, clonotype counts were normalized to relative frequencies and diversity metrics recalculated after rarefaction to a uniform sequencing depth of more than 1.5 million reads per sample, the minimum observed across all samples passing quality control ensuring comparability across time points. For the purposes of unique clone enumeration, clonotypes were defined by unique CDR3 nucleotide sequences in combination with V and J gene usage. For TCR convergence analysis, convergent clonotypes were identified as groups of CDR3 sequences sharing identical amino acid sequences and V gene segments but encoded by two or more distinct nucleotide sequences (the defining feature of convergent rearrangement) (6–8).

In dynamic immune repertoire analysis, the number and distribution of unique clones reflect the overall diversity of the adaptive immune response. An increase in unique clones suggests expansion of diverse lymphocyte populations, while a decrease may indicate clonal contraction or dominance of specific tumor-reactive clones.

Raw TCRβ sequencing data are publicly accessible at NCBI Sequence Read Archive under BioProject accession PRJNA1379651 (https://www.ncbi.nlm.nih.gov/sra/PRJNA1379651).

The experimental protocol was approved by the Human Research Ethics Committee of Ramathibodi Hospital, Mahidol University, Bangkok, Thailand (Institutional Review Board number COA. MURA2021/956). All methods were performed in accordance with relevant guidelines and local regulations.

### Treatment regimens

First-line platinum-based chemotherapy regimens were administered according to standard institutional protocols.

1. Carboplatin plus Paclitaxel: Paclitaxel 175 mg/m² followed by Carboplatin at an area under the concentration-time curve (AUC) of 5, administered on Day 1 of a 21-day cycle. Standard antiemetic and hypersensitivity premedication—comprising dexamethasone, H1 and H2 receptor antagonists, and a 5-HT3 receptor antagonist—was administered prior to each infusion.
2. Carboplatin plus Pemetrexed (non-squamous histology): Pemetrexed 500 mg/m² and Carboplatin AUC 5 on Day 1 of a 21-day cycle. Folic acid supplementation (350–1000 mcg orally once daily) and intramuscular vitamin B12 (1000 mcg every nine weeks) were administered per standard protocol to attenuate pemetrexed-associated haematologic toxicity.
3. Carboplatin plus Etoposide: Carboplatin AUC 5 on Day 1, combined with Etoposide 100 mg/m² on Days 1, 2, and 3 of a 21-day cycle. Carboplatin dosing was calculated using the Calvert formula: Dose (mg) = Target AUC × (GFR + 25).
4. Carboplatin plus Gemcitabine: Carboplatin AUC 5 on Day 1, with Gemcitabine 1000–1100 mg/m² on Days 1 and 8 of a 21-day cycle. Dose modifications were applied for Grade 3 or 4 thrombocytopenia according to protocol.
5. Gemcitabine monotherapy (administered when platinum-based doublet therapy was contraindicated): Gemcitabine 1000 mg/m² on Days 1, 8, and 15 of a 28-day cycle, followed by a seven-day recovery interval.

### Statistical analysis

Baseline characteristics, tumor features, clinical outcomes, and adverse events were summarized using descriptive statistics. The normality of continuous data was assessed using the Shapiro–Wilk test. Based on the distribution, parametric tests (Pearson’s correlation) were applied to normally distributed data, whereas non-parametric tests (Wilcoxon rank-sum test) were used for non-normally distributed data.

T-cell receptor (TCR) repertoire metrics were analyzed across individual patients and pooled samples. Shannon diversity was calculated to quantify TCR clone diversity, with higher values indicating greater diversity. Correlations between Shannon diversity and disease status were assessed using Pearson’s correlation. TCR convergence was defined as the cumulative frequency (dimensionless proportion of total sequencing reads, range 0–1) of TCRβ clonotypes sharing an identical CDR3 amino acid sequence and V gene segment, encoded by two or more distinct nucleotide sequences. This metric serves as a surrogate for antigen-driven, convergent clonal selection. TCR evenness was used to reflect the relative abundance distribution among TCR clones. Because immune repertoire alterations may occur at the time of disease progression (Time Point 3), only Time Points 1 and 2 were included in the pooled statistical analyses, whereas Time Point 3 was analyzed at the individual patient level.

Downstream analyses were performed using RStudio (version 2024, build 12.1 + 563) to compare changes in TCR convergence, evenness, and diversity between the progressive disease (PD) and disease hematologic control group (DCG). Spearman rank correlation (ρ) was used to assess monotonic associations between continuous variables, specifically TCR convergence frequency and treatment time point in the disease control group. Group comparisons of continuous repertoire metrics between PD and disease control patients were performed using the Wilcoxon rank-sum test (Mann–Whitney U test). To account for multiple comparisons across the three primary inferential tests, the Benjamini–Hochberg false discovery rate (BH-FDR) procedure was applied; BH-adjusted q-values are reported alongside raw p-values for each test. A p-value < 0.05 and a BH-adjusted q-value < 0.05 were considered statistically significant. Overall survival was estimated using the Kaplan–Meier method and groups were compared using the log-rank test.

## Results

### Baseline characteristics

**Figure 2:**
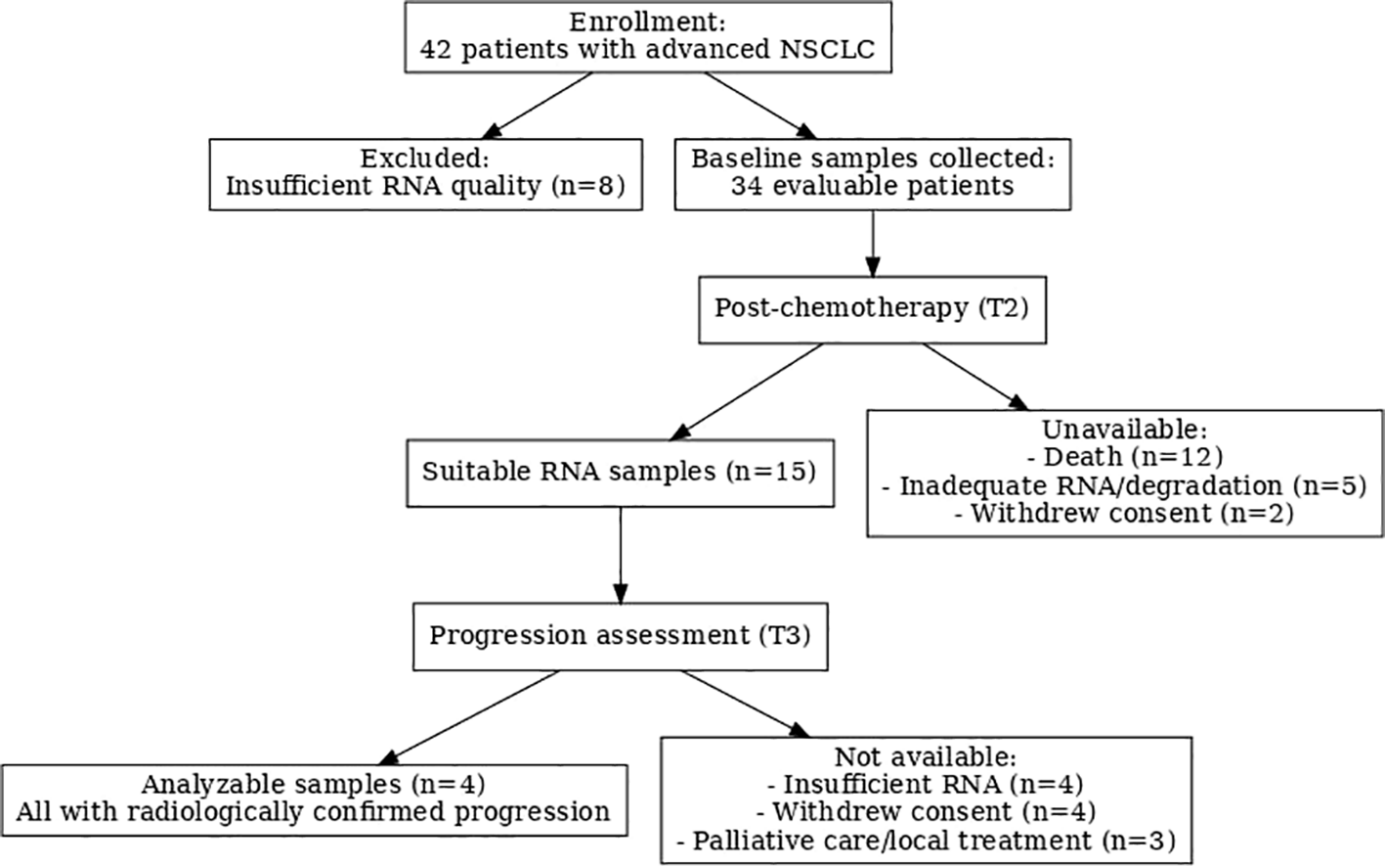
CONSORT diagram of patient enrollment and sample availability.

A total of 42 patients with advanced non–small cell lung cancer (NSCLC) were enrolled for baseline blood collection. Eight were excluded due to insufficient RNA quality, resulting in 34 evaluable cases with successfully obtained baseline samples. At the post-chemotherapy assessment (Time point 2), 15 samples remained suitable for analysis; the remainder were unavailable due to patient death (n=12), inadequate RNA yield or sample degradation (n=5), or withdrawal of consent (n=2). By the third assessment (Time point 3), additional attrition occurred, including insufficient RNA quality (n=4), withdrawal of consent (n=4), and transition to palliative care or local treatment (n=3), leaving four patients with analyzable samples. All four demonstrated radiologically confirmed disease progression following completion of chemotherapy **Figure 2**.

Three patients enrolled in clinical trials received investigational platinum-based cytotoxic doublet regimens without immunotherapy components, as confirmed by protocol review; none received immunomodulatory biologics, targeted agents, or checkpoint inhibitors as part of their investigational treatment. The OS-evaluable population comprised 28 patients (the 34 baseline-evaluable patients exclude 6 who withdrew consent at T2 [n = 2] or T3 [n = 4]); patients excluded from blood-based analyses due to death prior to sampling or insufficient RNA quality were retained in the OS analysis because survival data were available.

The median age was 66.3 years (SD ± 9.8), and the majority were male (59%). The mean height and weight were 161.9 cm (SD ± 13.2) and 61.8 kg (SD ± 13.2), respectively. Regarding smoking status, 59% were current or former smokers, while 41% had never smoked. Common comorbidities included dyslipidemia (44%), diabetes mellitus (21%), and hypertension (50%). Performance status, assessed by Eastern Cooperative Oncology Group (ECOG) criteria, showed that 15% of patients had ECOG 0, 44% had ECOG 1, 23% had ECOG 2, and 18% had unknown status. Histologically, adenocarcinoma was the predominant subtype (79%), followed by squamous cell carcinoma (12%) and other histologies (9%). According to the 8th AJCC staging, 18% of patients were stage III, 59% were stage IVA, and 23% were stage IVB at diagnosis. Metastatic involvement at baseline was most commonly observed in the lung (38%), pleura (35%), liver (18%), bone (18%), and brain (9%) (**Table 1**).

**Table 1:** Baseline Characteristics.

Characteristics	Time point1; N = 34	Time point2; N = 15
	N (%)	N (%)
Gender		
Male	20 (59)	11 (73)
Female	14 (41)	4 (27)
**Age** (years, mean ± SD)	66.3 ± 9.8	69.5 ± 8.0
**Height** (cm, mean ± SD)	161.9 ± 13.2	159.5 ± 16.2
**Weight** (kg, mean ± SD)	61.8 ± 13.2	57.1 ± 12.5
Smoking status		
Current/former smoker	20 (59)	11 (73)
Never smoker	14 (41)	4 (27)
Comorbidities		
Dyslipidemia		
No	19 (56)	5 (33)
Yes	15 (44)	10 (67)
Diabetes mellitus		
No	27 (79)	11 (73)
Yes	7 (21)	4 (27)
Hypertension		
No	17 (50)	3 (20)
Yes	17 (50)	12 (80)
ECOG PS		
0	5 (15)	1 (7)
1	15 (44)	7 (47)
2	8 (23)	5 (33)
unknown	6 (18)	2 (13)
Histology		
Adenocarcinoma	27 (79)	8 (53)
Squamous cell carcinoma	4(12)	3 (20)
Others	3(9)	4 (27)
Staging		
III	6(18)	2 (13)
IVA	20(59)	13 (87)
IVB	8(23)	0 (0)
Distant metastatic site		
Lung		
No	21(62)	12 (80)
Yes	13(38)	3 (20)
Pleura		
No	22(65)	11 (73)
Yes	12(35)	4 (27)
Bone		
No	28(82)	13 (87)
Yes	6(18)	2 (13)
Brain		
No	31(91)	14 (93)
Yes	3(9)	1 (7)
Liver		
No	28(82)	13 (87)
Yes	6(18)	2 (13)
PDL1 (IHC by 22C3)		
<1%	6(18)	4 (27)
1-49%	8(24)	4 (27)
≥50%	11(32)	4 (27)
Unknown	9(26)	3 (20)
First line treatment		
Platinum doublet chemotherapy	25(73)	14 (93)
Clinical trial chemotherapy	3(9)	0 (0)
Other chemotherapy	3(9)	1 (7)
No treatment (Supportive care)	3(9)	0 (0)
**Median cycle of first line treatment**	5	5

### Molecular and biomarker profile

The majority of patients were EGFR wild-type (82%), with 6% harboring EGFR mutations (EGFR mutations detected were non-sensitizing variants ineligible for approved EGFR-targeted therapies) and 12% with unknown EGFR status. ALK rearrangements were identified in 3% of patients, while 24% had unknown ALK status. ROS1 immunohistochemistry (IHC) was negative in 41%, positive in 3%, and unknown in 56%. BRAF mutations were identified in only one patient (3%), while 94% had unknown BRAF status.

PD-L1 expression, assessed using the 22C3 antibody clone, was <1% in 18%, 1–49% in 24%, and ≥50% in 32%, with 26% of cases lacking PD-L1 results.

### First-line treatment

Most patients (73%) received platinum-based doublet chemotherapy as first-line treatment. The remainder either participated in clinical trials (9%), received other treatments (9%), or were not treated (9%).

### Disease response

Among the 15 patients evaluable at T2, disease control was achieved in 11 cases (73%), comprising 4 patients (27%) with partial response (PR) and 7 patients (46%) with stable disease (SD); together constituting the Disease Control Group (DCG). Progressive disease was observed in 4 patients (27%) (**Table 2**).

**Table 2:** Treatment response and disease status in patients with advanced NSCLC after chemotherapy.

Disease status	N	%
Disease Control Group Partial response (PR) Stable disease (SD)	**11** 4 7	**73** 27 46
Progressive disease	**4**	**27**

### Changing of immune repertoire and clinical correlation in pre and post chemotherapy period (Time Points 1 and 2)

**Figure 3:**
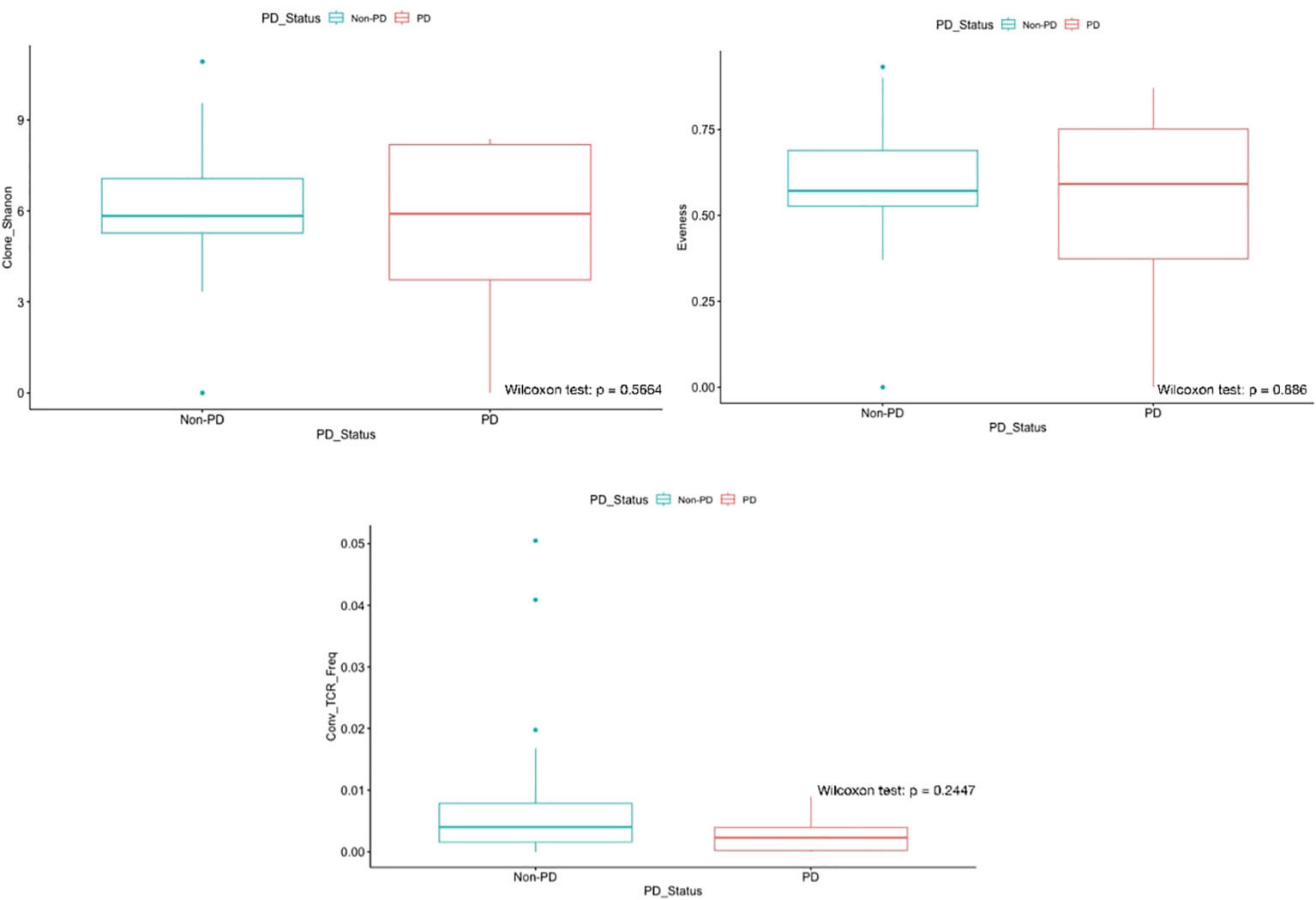
Box-and-whisker plots of pooled analyses for Shannon diversity, Evenness, and T-cell receptor (TCR) convergence frequency, stratified by disease status. No statistically significant difference was observed between groups for Shannon diversity or evenness (Spearman test, p > 0.05 for both). TCR convergence frequency was higher in the non-PD group (0.0040 vs. 0.0023; Spearman test, p > 0.8169).

Shannon diversity was slightly higher and more variable in the PD group (5.91) compared with the disease control group (5.84), although the difference did not reach statistical significance. This trend suggests greater heterogeneity of the T-cell repertoire among patients with progressive disease, whereas the relatively lower diversity in the disease control group may reflect a more focused clonal response directed against tumor neoantigens despite ongoing chemotherapy. Consistent with this observation, no significant differences in evenness were observed between PD and disease control groups (0.59 and 0.57, respectively). Higher evenness typically indicates a repertoire composed of numerous clones with relatively balanced frequencies, whereas lower evenness reflects preferential expansion of dominant clones **Figure 3**.

TCR convergence frequency, defined as the aggregate frequency of TCRβ clones sharing both a variable gene and an identical CDR3 amino acid sequence within a sample, was greater in the disease control cohort (0.0040 vs. 0.0023 in PD). This finding suggests that increased convergence may represent tumor-specific clonal expansion associated with disease control **Figure 3**.

**Figure 4:**
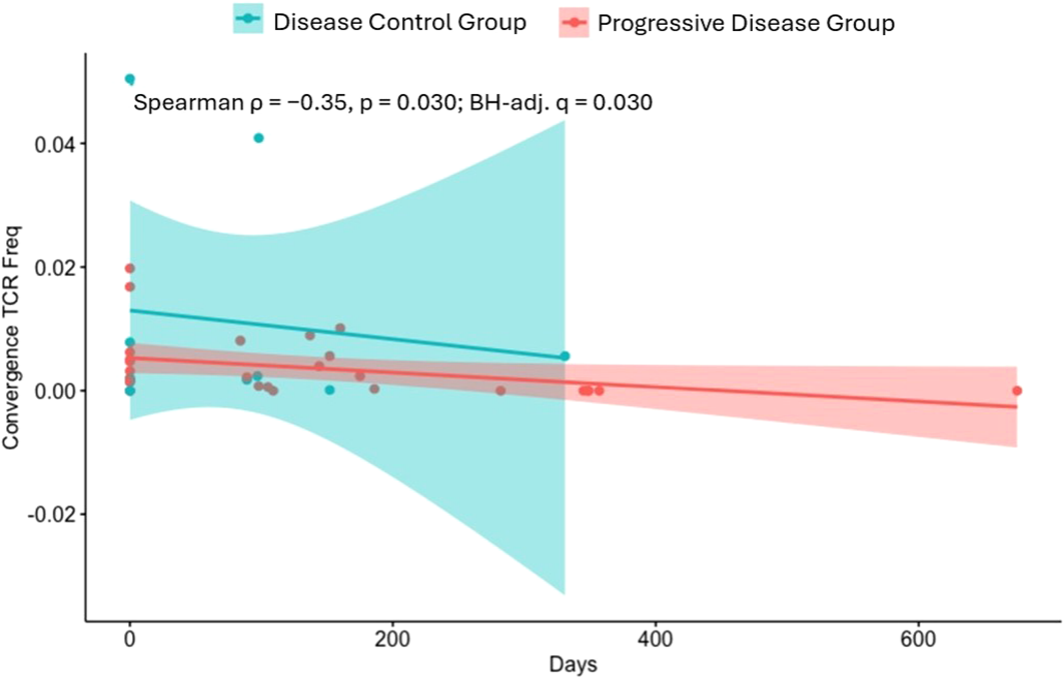
Scatterplot indicating relationship between TCR convergence frequency over treatment time points, stratified by disease status (PD vs disease control). Spearman correlation ρ = −0.35, p = 0.030; BH-adj. q = 0.030), PD, progressive disease; TCR, T-cell receptor.

A pooled analysis across all patients demonstrated that in the disease control group, shown in blue, convergent TCR frequency exhibited a negative correlation with time (Spearman ρ = −0.35, p = 0.030; BH-adj. q = 0.030), indicating a gradual decline over the follow-up period, **Figure 4**. In contrast, the PD group, shown in red, maintained consistently low levels of convergent TCR frequency without significant temporal change. The decreasing trend observed in the disease control group may reflect a dynamic yet initially robust immune response that diminishes with prolonged treatment exposure, whereas the persistently low levels in the PD group suggest insufficient or ineffective clonal expansion, consistent with ongoing disease progression.

**Figure 5:**
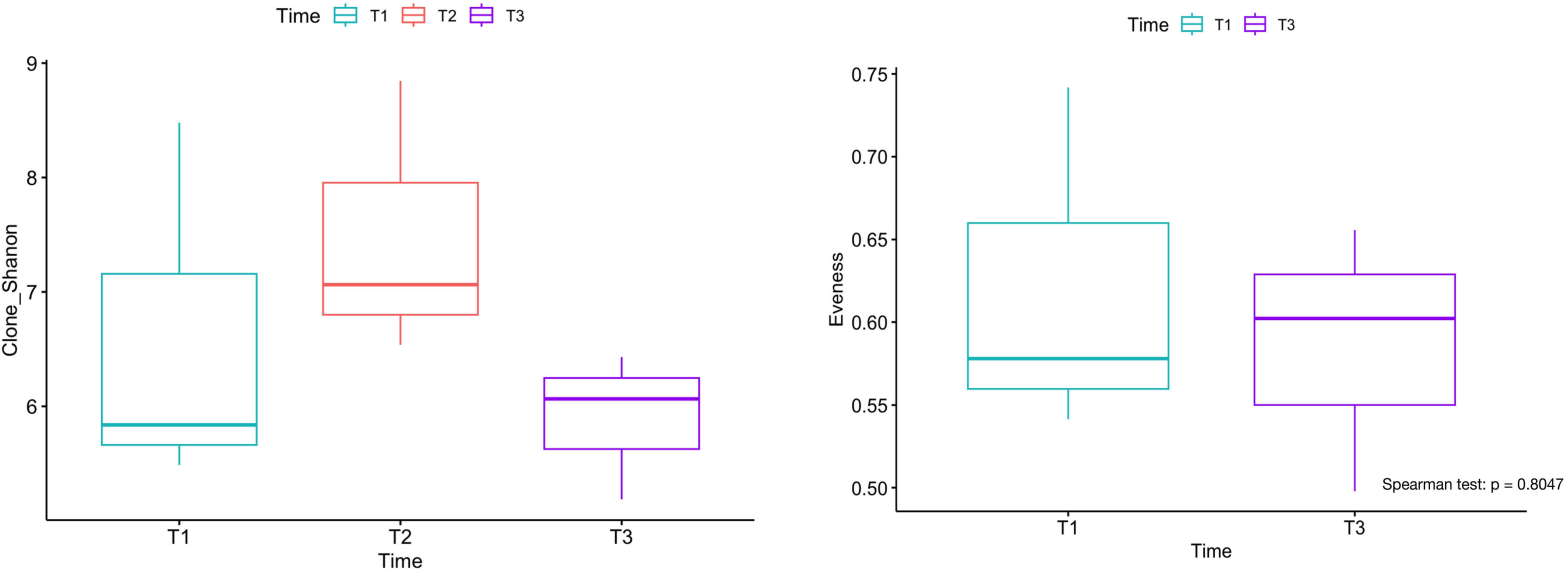
Dynamic changes in the T-cell receptor (TCR) repertoire and Shannon diversity across treatment time points in patients with progressive disease.

In a timepoint-specific analysis of the progressive disease (PD) cohort, we observed dynamic changes in the T-cell receptor (TCR) repertoire. Shannon diversity increased following completion of chemotherapy (T1 = 5.84, T2 = 7.06), accompanied by a rise in TCRβ convergence frequency (T1 = 0.0032, T2 = 0.0101), suggesting the transient expansion of a diverse, tumor-reactive T-cell population. Only 4 patients was provides samples at all three time points by the subsequent assessment (T3: Shannon6.06, TCR convergence frequency0.0069),both Shannon diversity and TCRβ convergence frequency declined, reflecting a loss of immune repertoire diversity and clonal convergence, **Figure 5**. In this small descriptive cohort, this pattern is consistent with T-cell exhaustion, clonal contraction, or impaired antigen presentation, all of which have been associated with diminished tumor recognition in larger published series. These observations derived from four patients and therefore intended as descriptive and hypothesis-generating only raise the possibility that such immune attrition may contribute to disease progression despite initial treatment-induced activation. These findings highlight the transient nature of chemotherapy-associated immune activation in advanced NSCLC and suggest that strategies aimed at sustaining or re-invigorating T-cell responses such as checkpoint blockade, costimulatory agonists, or novel immunomodulatory combinations may be required to achieve durable disease control.

**Figure 6:**
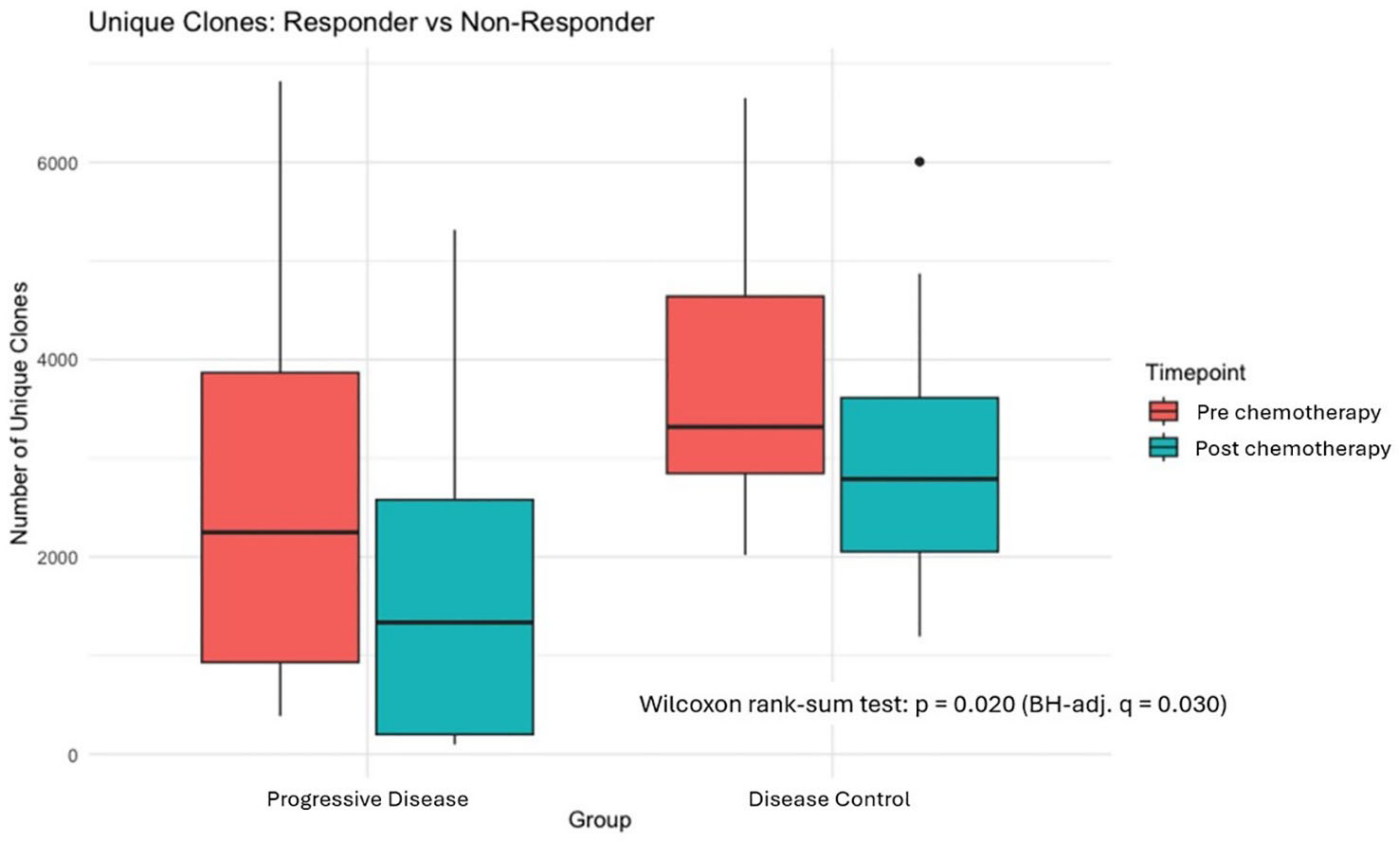
Box-and-whisker plots depicting the number of unique TCRβ clones stratified by disease status (Disease Control Group [DCG] vs. Progressive Disease [PD]) and treatment time point (Pre: T1; Post: T2). The decline in clone counts was significantly greater in PD patients than in DCG patients (Wilcoxon rank-sum test on delta-clone counts: p = 0.020; BH-adj. q = 0.030). The outlier dot in the DCG Post group (~5,900 clones) represents a single patient and did not materially influence group statistics.

To further delineate the dynamics of T-cell immunity, we performed an in-depth analysis of TCRβ clonotypes stratified by treatment response at baseline and post chemotherapy. Consistent with prior reports on TCR convergence, we observed the decline in unique TCR clone counts from T1 to T2 was significantly greater in PD patients (median: 2,248 to 1,335; a reduction of ~41%) than in non-PD patients (Disease Control Group; median: 3,318 to 2,790; a reduction of ~16%), as assessed by Wilcoxon rank-sum test comparing delta-clone counts between groups (p = 0.020; BH-adj. q = 0.030), **Figure 6**. In contrast, patients without progression (Disease control group) demonstrated only a modest reduction in clonal diversity following treatment. Importantly, disease control group exhibited a higher median number of unique clones at baseline compared with progressive disease group, suggesting that greater pre-treatment T-cell repertoire diversity may be associated with improved clinical outcomes.

**Figure 7:**
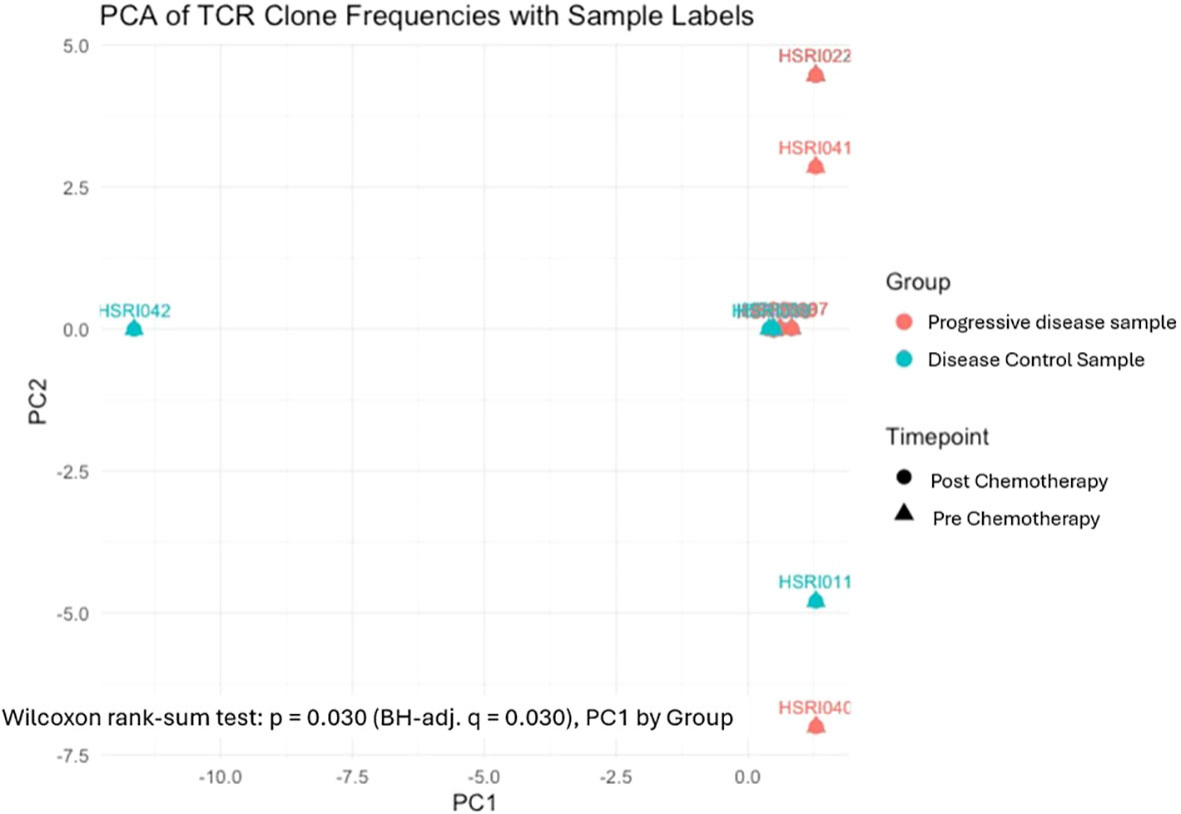
Principal component analysis (PCA) of TCRβ clone frequencies in patients with progressive disease (PD; orange, n = 4) and disease control (teal, n = 11), across pre- and post-chemotherapy time points. PD samples showed wide PC2 dispersion; disease control samples clustered centrally, with one post-chemotherapy outlier (HSRI042) displaced along the negative PC1 axis. Wilcoxon rank-sum test on PC1 scores by group: p = 0.030; BH-adj. q = 0.030.

Principal component analysis (PCA) of TCR clone frequencies demonstrated divergent repertoire organization between disease groups along both principal components (Wilcoxon rank-sum test on PC1 scores by group: p = 0.030, BH-adj. q = 0.030), **Figure 7**.

Progressive disease samples (orange) were distributed across a wide vertical range along the PC2 axis — spanning from HSRI040 at PC2 ≈ −7.0 through HSRI041 at PC2 ≈ +2.8 to HSRI022 at PC2 ≈ +4.8 — while remaining compressed near PC1 ≈ 0 reflects marked inter-individual heterogeneity in clonal composition among progressive disease (PD) patients, consistent with dysregulated or ineffective T-cell clonal expansion that varies substantially between patients despite a shared clinical outcome.

Disease control samples (teal) exhibited a qualitatively distinct pattern. The majority formed a tight central cluster near the origin (PC1 ≈ 0, PC2 ≈ 0), indicative of conserved and stable repertoire architecture across patients and treatment timepoints. Notably, one post-chemotherapy disease control sample (HSRI042) was displaced markedly along the negative PC1 axis (PC1 ≈ −10.5, PC2 ≈ 0), representing an individual with a substantially remodelled clonal frequency structure following treatment. This sample-level shift on PC1 may have contributed to the statistically significant PC1 separation between groups; however, given that the PD group comprised only four patients, all findings should be considered preliminary and hypothesis-generating only. A sensitivity analysis excluding HSRI042 was not feasible in this cohort but should be a priority in future studies with larger sample sizes.

Together, these findings suggest that chemotherapy-induced immune perturbations drive divergent patterns of TCR repertoire remodelling depending on clinical outcome. The vertical PC2 scatter in progressive disease samples reflects disordered, heterogeneous clonal dynamics, whereas the compact central clustering of most disease control samples punctuated by one patient’s directed PC1 shift may reflect preservation of tumor-reactive T-cell populations alongside selective post-treatment clonal reorganization. These results highlight repertoire organization as a potential biomarker of therapeutic response in advanced NSCLC, and underscore that PCA captures clinically relevant immune states not detectable by univariate diversity metrics alone.

**Figure 8:**
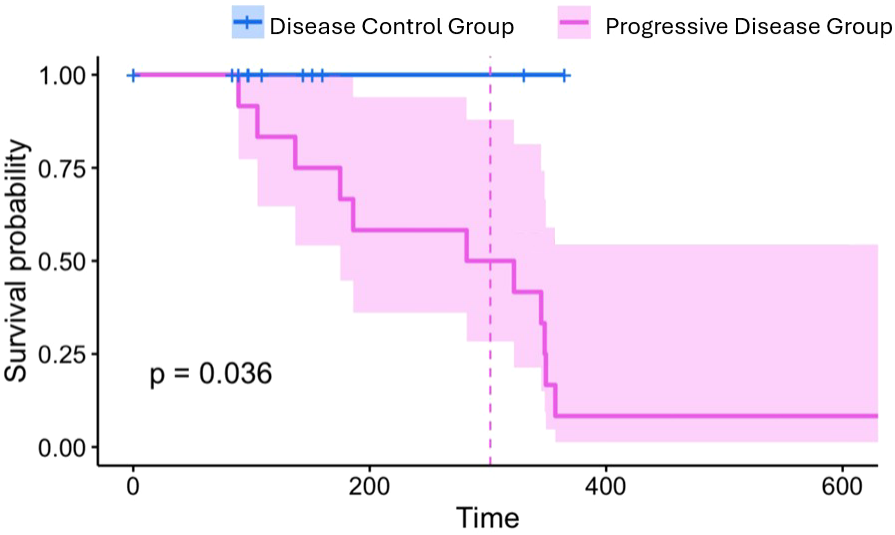
Kaplan–Meier analysis of overall survival (OS) stratified by disease status at T2 (Disease Control Group [DCG] vs. Progressive Disease [PD]). OS was analysed in 28 patients (34 baseline-evaluable exclude 6 consent withdrawals).Log-rank test, p = 0.036. OS, overall survival; DCG, disease control group; PD, progressive disease.

The Kaplan–Meier analysis demonstrated a separation in survival probability between patients with progressive disease (PD) and those without progression (Disease control group). All patients in the disease control group maintained a 100% survival probability throughout the observation period (median follow-up: 302 days). By contrast, the PD group exhibited a stepwise decline in survival, with the probability decreasing to approximately 50% after 200 days and showing a further reduction beyond 400 days. The difference in overall survival between groups reached statistical significance (log-rank test, p = 0.036; **Figure 8**).

## Discussion

Historically, the management of advanced NSCLC has relied primarily on cytotoxic chemotherapy, which provided modest survival benefits and was often associated with substantial toxicity (3). As highlighted by Molina et al., long-term outcomes for patients without targetable alterations were historically poor, with median survival rarely exceeding one year despite treatment advances during that era (3). Our findings expand upon this foundation by showing that even within the context of conventional chemotherapy, distinct immune repertoire dynamics emerge between patients who achieve disease control and those who progress. These observations suggest that immune repertoire dynamics may influence clinical outcomes even in the context of conventional cytotoxic chemotherapy, predating the era of immune checkpoint inhibitors.

The findings demonstrate that chemotherapy-induced TCR repertoire dynamics differ systematically between patients who achieve disease control and those who progress, with stable clonal convergence and preserved unique clone counts emerging as potential discriminators of clinical outcome.

Our results provide exploratory evidence that chemotherapy in non-targetable advanced NSCLC transiently reshapes the TCR repertoire, highlighting immune activation followed by attrition, with distinct patterns observed between patients who achieved disease control (Disease Control Group) and those with progressive disease (PD). Our findings demonstrate that Disease control patients maintained a more convergent repertoire, suggestive of tumor-specific clonal expansion, while PD patients exhibited greater repertoire heterogeneity and transient immune activation that diminished over time. These results highlight the prognostic potential of immune repertoire dynamics in a patient population where effective biomarkers remain limited. Furthermore, the pronounced contraction of unique TCR clone counts observed in PD patients (median: 2,248 to 1,335 clonotypes; p = 0.020, BH-adj. q = 0.030) relative to the modest reduction in non-PD patients (median: 3,318 to 2,790) is consistent with chemotherapy-induced lymphodepletion selectively eroding the breadth of antigen-reactive T-cell populations in those lacking an effective antitumor immune response. The preservation of clonal diversity in disease control patients may reflect homeostatic replenishment of functional T-cell populations or the selective expansion of tumor-reactive clones that partially offsets chemotherapy-mediated lymphodepletion.

The observation that disease control patients demonstrated higher TCR convergence and more stable repertoire architecture aligns with prior studies in NSCLC treated with immunotherapy. Iemwimangsa et al. (11) reported that increased TCR convergence was associated with favorable outcomes during durvalumab consolidation, supporting the hypothesis that clonal expansion of tumor-reactive T cells underlies effective disease control. Similarly, Reuben et al. (10) showed that breadth and clonality of the TCR repertoire were correlated with improved survival, underscoring the relevance of repertoire-focused biomarkers across therapeutic contexts. Although our cohort did not receive immunotherapy, the parallel patterns suggest that chemotherapy may also exert immunomodulatory effects capable of shaping T-cell responses, albeit transiently.

To contextualize the biological plausibility of our findings, baseline TCR repertoire metrics observed in the present cohort were compared against reference ranges reported in independent published studies. With respect to Shannon diversity, our baseline values (disease control: 5.84; PD: 5.91) fall within the range of 5.7–6.4 reported by Reuben et al. (10) in peripheral blood of NSCLC patients across disease stages, affirming that our cohort exhibits repertoire diversity consistent with established NSCLC populations. Regarding convergence frequency, our observed baseline values (0.0023–0.0040) closely overlap with the range of 0.002–0.006 reported by Iemwimangsa et al.11 in stage III NSCLC patients prior to durvalumab consolidation, lending further biological plausibility to our metric ranges. In contrast, population-level immunosequencing data from healthy adult donors reported by Emerson et al. (14) and Qi et al. indicate that peripheral blood TCR repertoires in healthy individuals typically display Shannon diversity indices of 6.0–8.0 and convergence frequencies below 0.001 (15). The modestly reduced diversity and elevated convergence observed in our NSCLC cohort relative to these healthy donor benchmarks are directionally consistent with antigen-driven clonal contraction in the setting of malignancy. We acknowledge, however, that direct quantitative comparisons across studies are inherently constrained by differences in sequencing platform, sequencing depth, CDR3 definition, and normalisation strategy, and results should therefore be interpreted with appropriate caution.

The transient rise in both Shannon diversity and convergence among PD patients post-chemotherapy, followed by subsequent decline, may reflect an initial wave of T-cell priming that was not sustained. This phenomenon is consistent with reports of chemotherapy-induced immunogenic cell death, which can transiently expand the T-cell repertoire but may also lead to T-cell exhaustion or impaired antigen presentation over time (10, 12, 18–20). Overall survival was analysed in 28 patients (those who did not withdraw consent), irrespective of blood sample availability. Within the T2 response-evaluable subset, disease control patients maintained 100% survival probability throughout the observation period (median follow-up: 302 days), whereas PD patients exhibited a stepwise decline reaching approximately 50% by 200 days and further attrition beyond 400 days (log-rank p = 0.036). This statistically significant separation in overall survival adds prognostic weight to the observed immune repertoire differences between groups. We note that the observation period is short (median follow-up 302 days) and survival data for the DCG are likely immature; these estimates should be interpreted with caution.

When considered alongside more recent studies (10–12, 18–21), our results suggest that chemotherapy may exert not only cytotoxic but also immunomodulatory effects, shaping T-cell repertoire organization in ways that can influence prognosis. This perspective bridges historical treatment paradigms with emerging concepts in immuno-oncology, highlighting the continued importance of immune profiling in non-targetable advanced NSCLC and reinforcing the rationale for integrating repertoire-based biomarkers into modern treatment paradigms.

In addition, the greater heterogeneity of repertoire composition observed in PD patients through principal component analysis (PCA) may indicate ineffective or dysregulated T-cell clonal responses. Comparable findings were described in studies of immune checkpoint inhibitors therapy, where increased repertoire entropy and lack of dominant clonal expansion correlated with inferior outcomes (10, 12, 15). Together, these results suggest that repertoire organization—beyond conventional diversity metrics—may provide clinically relevant insights into treatment responsiveness.

Importantly, our results extend the understanding of immune repertoire dynamics to a population of non-targetable advanced NSCLC patients, who represent a therapeutic challenge due to the absence of effective targeted therapies. While most prior work has focused on immunotherapy-treated cohorts, our study demonstrates that repertoire-based biomarkers may also be informative in chemotherapy-treated patients, offering opportunities for broader clinical application.

This study has several limitations. First, the sample size was relatively small, which may have limited statistical power to detect differences in certain repertoire metrics. Second, substantial longitudinal attrition from 42 patients at baseline to 15 at T2 and 4 at T3 introduced survival and selection bias that warrants careful consideration. The principal driver of attrition was death prior to T2 sampling: 74% of patients excluded from T2 analysis had died by the data cut-off, compared with 27% of those with evaluable T2 samples (p = 0.014). Formal comparison of baseline characteristics between T2-included (N = 15) and T2-excluded (N = 19) patients revealed no statistically significant differences in age (mean 69.5 vs. 63.8 years; p = 0.104), sex (p = 0.171), or smoking status (p = 0.171); however, statistically significant differences in disease stage (0% vs. 42% stage IVB; p = 0.005) and histology (adenocarcinoma 53% vs. 100%; p = 0.011) were identified, with the T2-excluded group being enriched for more advanced stage disease and uniform adenocarcinoma histology. These findings indicate that the longitudinally analysed cohort represents a biologically selected subset enriched for patients with sufficiently favourable disease trajectories to survive chemotherapy completion, as evidenced by significant differences in disease stage and histology distribution between those who did and did not provide T2 samples, and that longitudinal repertoire findings cannot be straightforwardly generalised to the broader patient population (**Supplementary Table 1**). Third, peripheral blood-derived TCR repertoires may not fully reflect the clonal architecture within the tumor microenvironment. Tumor-infiltrating lymphocytes (TILs) are enriched for antigen-experienced, clonally expanded populations and typically exhibit lower diversity and higher convergence than matched circulating T cells. While prior studies in NSCLC have demonstrated partial overlap between peripheral and intratumoral TCR repertoires, supporting the prognostic utility of blood-based profiling (10), direct paired tumor-blood comparisons were not feasible in the current palliative-intent cohort. Future studies incorporating paired tissue and liquid biopsy approaches will be essential to determine the degree to which peripheral repertoire dynamics mirror intratumoral immune states. Fourth, the lack of an immunotherapy-treated comparator group precludes direct conclusions on how chemotherapy versus immunotherapy-driven repertoire changes differ in magnitude or durability. Finally, PD-L1 expression data were unavailable in 26% of patients (9/34), limiting our ability to explore the relationship between PD-L1 status, TCR repertoire metrics, and treatment response. Future prospective studies should mandate PD-L1 assessment at enrollment to enable subgroup analyses stratified by immune checkpoint expression.

Despite these limitations, our findings underscore the importance of immune repertoire profiling in advanced NSCLC. Future studies should incorporate larger cohorts, longitudinal sampling, and integration with tumor immune microenvironment profiling to validate the predictive utility of repertoire metrics. Moreover, combining chemotherapy with immunotherapy may provide an avenue to sustain beneficial clonal expansions, warranting further clinical exploration. (6, 9).

Future studies should aim to expand beyond bulk TCR repertoire sequencing to more integrative approaches. Liquid biopsy–based profiling may enable real-time, minimally invasive monitoring of immune repertoire dynamics, providing longitudinal insights into clonal expansion or attrition during systemic therapy. Single-cell TCR sequencing can further delineate the transcriptional states and functional phenotypes of clonotypes, offering a direct link between repertoire architecture and effector function. In addition, spatial profiling of tumor tissue and tertiary lymphoid structures will help contextualize peripheral repertoire changes within the tumor microenvironment, clarifying whether observed systemic shifts mirror intratumoral immunity. Together, these technologies can advance the field toward multi-modal biomarkers that not only predict prognosis but also inform optimal sequencing or combination of chemotherapy and immunotherapy in advanced NSCLC.

## Data Availability

The datasets presented in this study can be found in online repositories. The names of the repository/repositories and accession number(s) can be found in the article/**Supplementary Material**.
