# Supplementary material for: Dynamics of the immune repertoire in recurrent, locally advanced NSCLC not amenable for definitive therapy and in stage IV disease receiving first-line chemotherapy
Source: Front Oncol. 2026 Apr 13;16:1787457. doi: 10.3389/fonc.2026.1787457 (PMC13111021; doi:10.3389/fonc.2026.1787457)
Supplement: Supplementary file 1 [file Table1.docx]

**Supplementary Table S1: Baseline Characteristics of T2-Included vs. T2-Excluded Patients**

| **Characteristic** | **T2 Included N = 15**  **N (%)** | **T2 Excluded N = 19**  **N (%)** | **p-value** |
| --- | --- | --- | --- |
| **Age**, years (mean ± SD) (median [I) | 69.5 ± 8.0 | 63.8 ± 11.9 | 0.104 |
| **Sex**, Male n (%) | 11 (73%) | 9 (47%) | 0.171*†* |
| **ECOG PS**, n (%) |  |  |  |
| 0 | 1 (7%) | 4 (21%) |  |
| 1 | 7 (47%) | 8 (42%) |  |
| 2 | 5 (33%) | 3 (16%) | 0.327* |
| Unknown | 2 (13%) | 4 (21%) |  |
| **Stage** (8th AJCC), n (%) |  |  |  |
| IIIA–IIIC | 2 (13%) | 4 (21%) |  |
| IVA | 13 (87%) | 7 (37%) |  |
| IVB | 0 (0%) | 8 (42%) | 0.005*‡* |
| **Histology**, n (%) |  |  |  |
| Adenocarcinoma | 8 (53%) | 19 (100%) ‡ | 0.011 |
| Squamous cell carcinoma | 3 (20%) | 1 (5%) |  |
| Other | 4 (27%) | 0 (0%) ‡ |  |
| **Smoking**, Ever n (%) | 11 (73%) | 9 (47%) | 0.171*†* |
| **Deceased at data cut-off**, n (%) | 4 (27%) | 14 (74%) | 0.014*†* |

** Formal chi-squared test excluding unknown ECOG PS; † Fisher exact test (two-sided); ‡* *Fisher exact test (IVA/B vs. other); Abbreviation: ECOG PS, Eastern Cooperative Oncology Group performance status.*
